# Optimizing DSA parameters for enhanced radiation safety in interventional surgery

**DOI:** 10.3389/fpubh.2025.1531240

**Published:** 2025-02-21

**Authors:** XunJin Zeng, Hao Wang, Guang Chen

**Affiliations:** Department of Radiological Intervention, Tianjin First Central Hospital, Nankai District, Tianjin, China

**Keywords:** interventional radiation, digital subtraction machine, radiation, dose, radiation protection

## Abstract

**Objective:**

The study aims to establish a reliable method for reducing radiation dose by analyzing variations in radiation dose from digital subtraction machines (DSA).

**Methods:**

The study investigates changes in bed plate height (80–110 cm), detection height (0–30 cm), visual field size (6 × 6 inches and 12 × 12 inches), and radiation doses affecting various body parts, including the lens, thyroid, chest, gonads, and lower limbs. Radiation doses were measured using Raysafe X2 dosimetry for patients and AT1123 meter for operators.

**Results:**

Compared to ordinary fluoroscopy, the low-dose fluoroscopy mode reduced the patient's radiation dose by 50.8% (from 13.2 to 6.5 mGy/min) and the operator's scattered radiation dose by 25–34% (lens dose reduced from 0.72 to 0.47 mGy). In photographic mode, the radiation dose was 3–4 times higher than in ordinary fluoroscopy (e.g., 53.9 vs. 13.2 mGy/min). Raising the bed plate height from 80 to 110 cm reduced the patient's direct radiation dose by 45.5% (from 24.2 to 13.2 mGy/min). The correct application of protective devices reduced the operator's scattering radiation by more than 10 times (e.g., gonads dose reduced from 4.07 to 0.41 mGy).

**Conclusion:**

Selecting the appropriate bed plate (90–100 cm) and detector height (10–20 cm), along with an optimal visual field (6 × 6 inches), can effectively reduce radiation doses for both patients and operators. The proper use of protective devices in peripheral interventional surgery is crucial for reducing scatter radiation, with reductions exceeding 90% in some cases.

## 1 Introduction

Fluoroscopy-guided interventional surgery has revolutionized modern medical practice by offering real-time imaging, minimally invasive procedures, and reduced recovery times compared to traditional surgical methods (1, 2), and corresponding damage, mainly radioactive skin damage mainly (3). However, the extensive use of fluoroscopy in interventional procedures has raised significant concerns regarding radiation exposure for both patients and operators. Patients undergoing interventional surgery are exposed to high levels of ionizing radiation, which can lead to deterministic effects such as skin injuries and stochastic effects like cancer risk (4, 5).

Similarly, operators, due to their proximity to the X-ray source, are exposed to scattered radiation, which can accumulate over time and lead to long-term health risks, particularly to the lens of the eye and the thyroid gland (6). Several studies have investigated strategies to mitigate radiation exposure during interventional procedures. For instance, low-dose fluoroscopy modes have been shown to reduce radiation doses significantly without compromising image quality (7, 8). Additionally, the use of protective equipment such as lead aprons, thyroid shields, and lead curtains has been widely adopted to minimize operator exposure (9, 10). However, the effectiveness of these measures can vary depending on the specific parameters of the digital subtraction angiography (DSA) machine, such as bed plate height, detector height, and field of view size.

Despite these advancements, there remains a gap in the literature regarding the systematic optimization of DSA parameters to achieve maximal radiation dose reduction while maintaining procedural efficacy. Previous studies have primarily focused on individual parameters, such as fluoroscopy mode or protective equipment, without considering the combined effects of multiple variables (11, 12). Moreover, the impact of bed plate height and detector height on radiation dose distribution has not been thoroughly explored, particularly in the context of peripheral interventional procedures.

This study aims to address these gaps by providing a comprehensive analysis of the effects of various DSA parameters, including bed plate height, detector height, fluoroscopy mode, and field of view size, on radiation dose for both patients and operators. By systematically evaluating these parameters, we seek to establish optimal settings that can significantly reduce radiation exposure without compromising the quality of interventional procedures. Furthermore, we investigate the efficacy of protective equipment in reducing scatter radiation, offering practical guidelines for enhancing radiation safety in clinical practice.

## 2 Materials and methods

### 2.1 Instruments and equipment

Measuring apparatus. Patient radiation was measured using Raysafe X2 dosimetry (range: 1 uGy/s-1 Gy/min) and operator radiation was measured using AT1123 meter (range: 50 nSv/h−10 Sv/h). Both devices were calibrated prior to the study using traceable standards from the National Institute of Metrology, China, ensuring accuracy and reliability of the measurements. The calibration process involved exposing the dosimeters to known radiation doses and comparing the readings to the reference values. The calibration certificates for both devices are available upon request.

DSA equipment. The test DSA device is a Shimadzu type Trinias 12 digital vascular subtraction machine. The DSA machine was calibrated according to the manufacturer's guidelines before the study, with particular attention to the tube voltage, tube current, and exposure time settings. The calibration was verified using a standard phantom to ensure consistent radiation output and image quality.

Die body 0.19 pieces of plexiglass (each with dimensions of 35 cm × 35 cm × 1 cm) were used to construct the patient direct-ray phantom. Plexiglass was chosen due to its radiological properties, which are similar to those of human soft tissue, particularly in terms of X-ray attenuation and scattering. The total thickness of 19 cm (achieved by stacking 19 pieces) was selected to simulate the average attenuation of X-rays through the human torso, which typically ranges from 15 to 20 cm for adults. The 35 × 35 cm size of each piece ensures full coverage of the typical field of view used in interventional procedures, allowing for accurate measurement of radiation doses. The phantom was validated by comparing the measured radiation doses to theoretical calculations based on the known properties of plexiglass and the DSA machine's output parameters. The results showed a high degree of agreement, confirming the suitability of the phantom for simulating patient radiation exposure.

**Figure 1:**
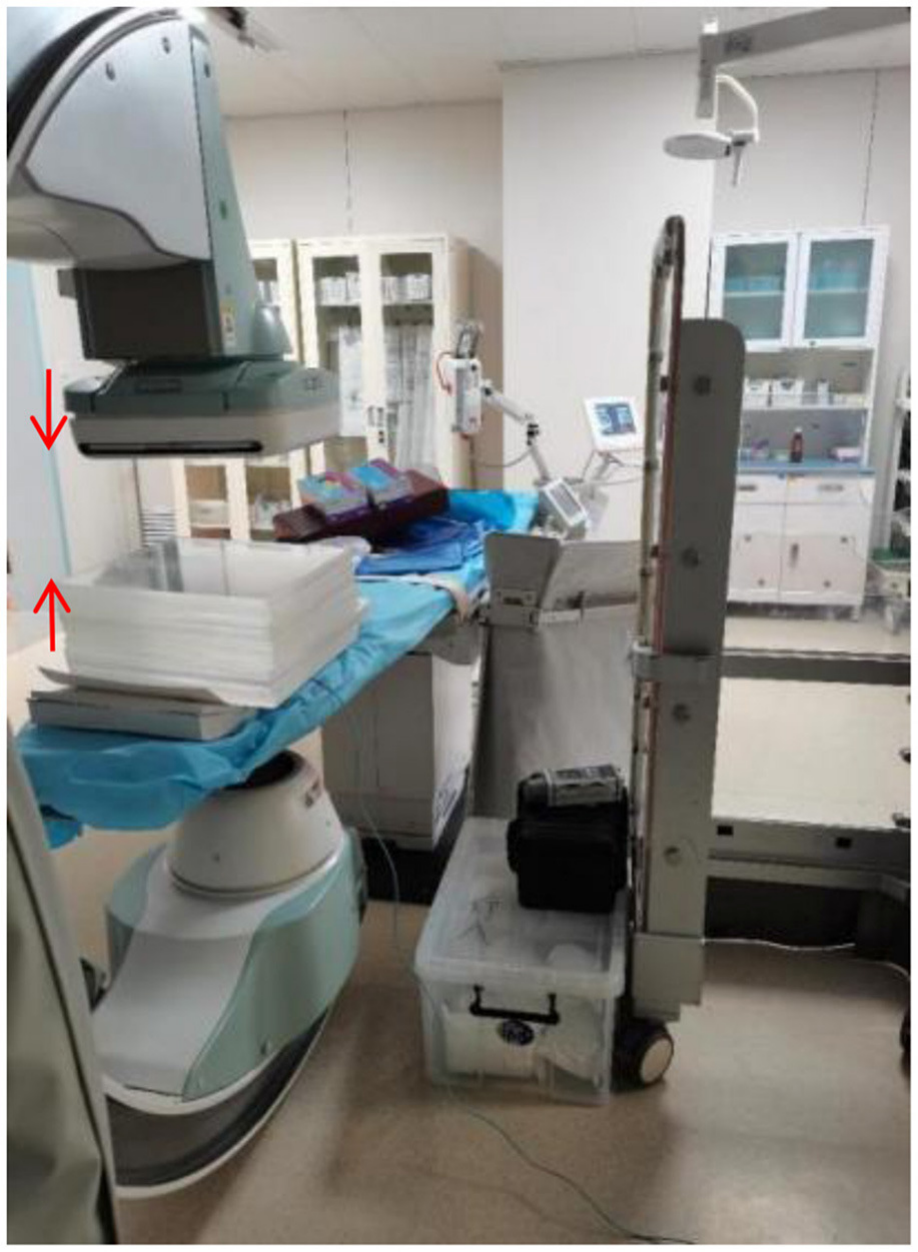
Position of the mold placement and measurement location for scattered radiation without protection. The phantom consists of 19 layers of plexiglass (35 cm × 35 cm × 1 cm each), simulating the attenuation properties of human soft tissue. The dosimeter probe is placed at the center of the field of view to ensure consistent and representative measurements. This setup is used to evaluate the baseline scattered radiation levels without any protective shielding.

Figure 1 illustrates the experimental setup for measuring scattered radiation without the use of protective devices. The plexiglass phantom, simulating patient tissue, is placed on the bed plate of the DSA machine. The dosimeter probe is positioned between the phantom and the bed plate to measure the scattered radiation. The setup ensures that the phantom fully occupies the field of view, and the dosimeter is centered within the imaging area. This configuration allows for accurate measurement of scattered radiation under controlled conditions.

**Figure 2:**
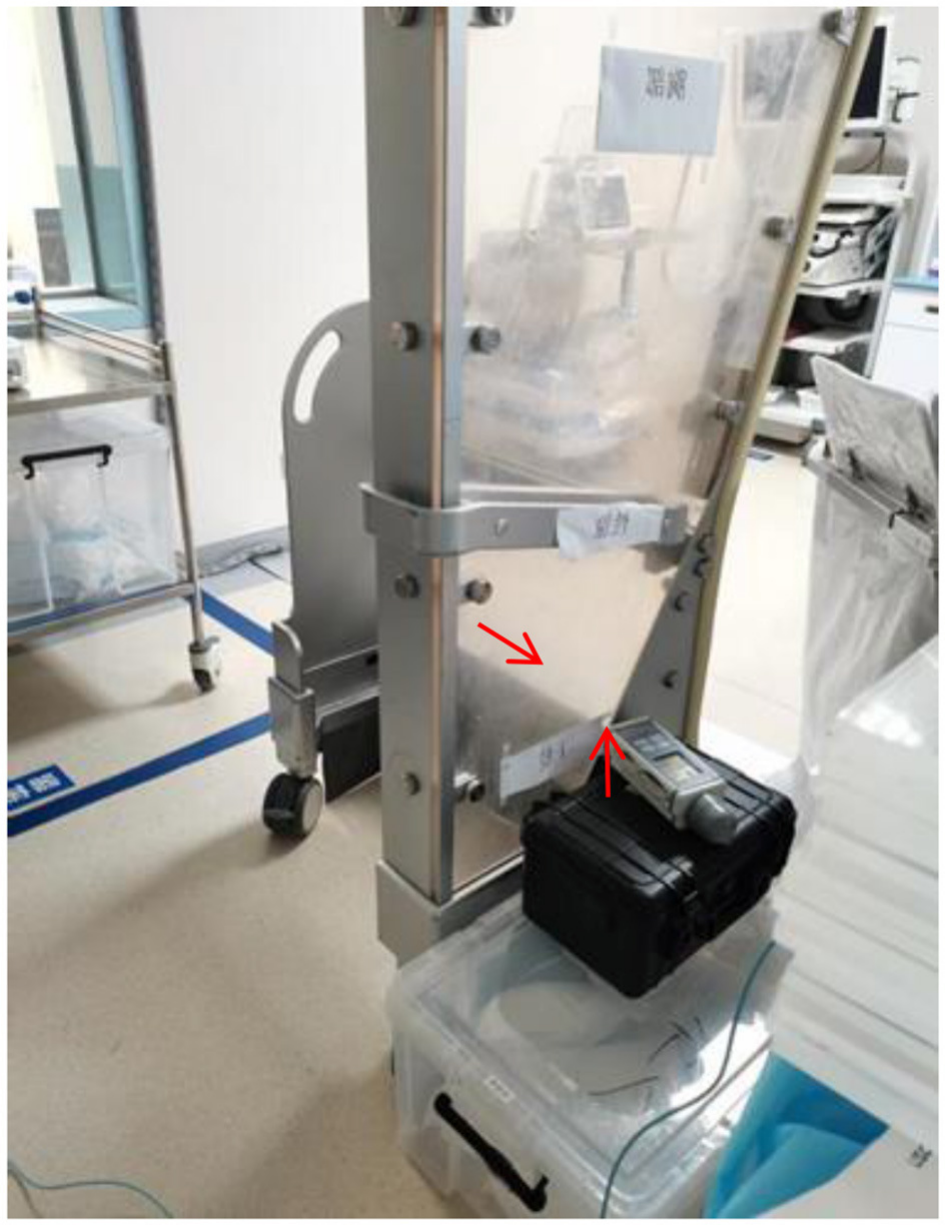
Measurement positions of scattering rays and placement of measuring instruments. The dosimeters are positioned at 40, 90, 120, 170, and 170 cm from the C-arm to represent the lower limb, gonad, thorax, thyroid, and lens, respectively. Measurements are taken under different fluoroscopy modes (normal and low-dose) to evaluate the impact of operational parameters on scattered radiation. This setup allows for a comprehensive assessment of scattered radiation distribution and its variation with changes in bed height, detector height, and field of view size.

Figure 2 depicts the measurement positions for scattered radiation and the placement of dosimeters during the experiment. The C-arm of the DSA machine is positioned at a fixed distance from the phantom, and dosimeters are placed at various heights (0, 10, 20, and 30 cm) to measure scattered radiation at different levels. The figure also shows the placement of dosimeters at specific anatomical locations (lens, thyroid, chest, gonads, and lower limbs) to assess operator radiation exposure.

### 2.2 Test method

Patient radiation dose testing. Plexiglass phantom is placed on the bed board, The dosimeter probe is placed between the mold body and the bed plate; Ensure that the harness limit is fully open, The phantom fills the entire field of vision and determines that the meter is located in the center of the field of vision. The selected mode is abdominal mode, which is commonly used in clinical practice for its balance between image quality and radiation dose. We adjusted the mode to pulsed fluoroscopy mode, pulse low dose fluoroscopy mode, and photography mode, respectively to evaluate the impact of different modes on radiation dose.

The frame rate was set at 15 fps, a standard setting in interventional procedures, as it provides sufficient temporal resolution for real-time imaging while minimizing radiation exposure. The cumulative number of collected frames was set to 150 frames to ensure a representative sample of radiation dose over a typical procedure duration.

To explore the effect of detector height on radiation dose, we adjusted the detector height to be infinitely close to the die body (0, 10, 20, and 30 cm). The bed plate height was varied at 80, 90, 100, and 110 cm from the ground to simulate different clinical scenarios. The vision size was chosen as 6 ^*^ 6 inches and 12 ^*^ 12 inches, representing small and large fields of view commonly used in interventional procedures.

Surgical operator radiation dose test. The measurement conditions are the same as the patient radiation dose test. The C arm is inserted by the physician and 60 cm from the C arm to measure the site scattering dose at 40, 90, 120, 17, and 170 cm were measured to represent the lower limb, gonad, thorax, thyroid and lens, respectively (6). Observe the scattered radiation test instrument data fluctuation when the data is stable or reaches the maximum value; the operator radiation dose only records the cumulative dose rate change before and after fluoroscopy 15 fps and 15 fps low mode.

## 3 Results

### 3.1 Radiation dose comparison in different modes

**Figure 3:**
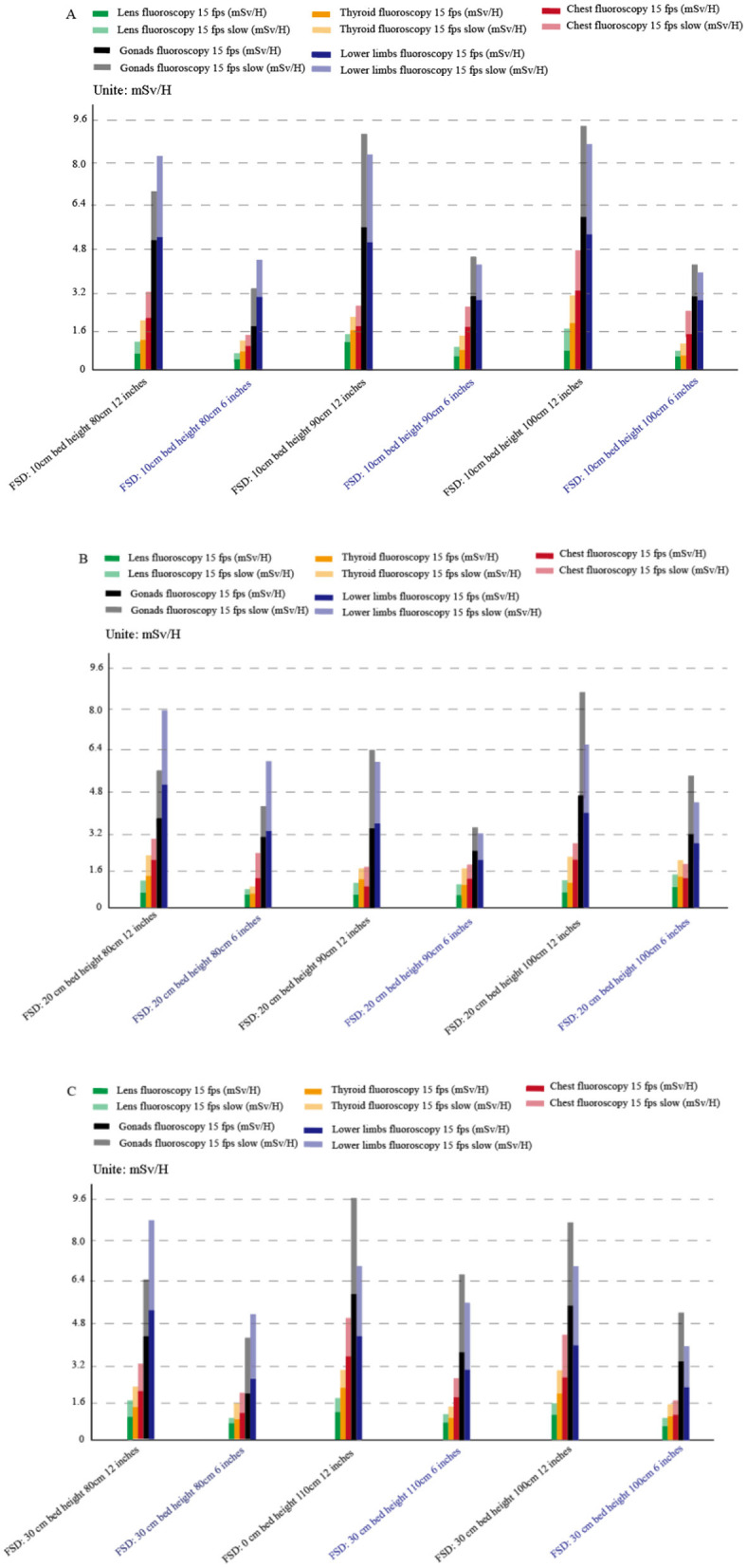
Operator radiation dose changes in normal fluoroscopy mode and low-dose fluoroscopy mode at different field sizes (**A**: FSD 10 cm; **B**: FSD 20 cm; **C**: FSD 30 cm). The blue coordinates represent the radiation dose under the 6-inch field, and the white coordinates represent the radiation dose under the 12-inch field. The figure illustrates the reduction in operator radiation dose when switching from normal fluoroscopy mode to low-dose fluoroscopy mode across different field sizes.

The change of fluoroscopy mode causes the change of several technical parameters, including the change of focus and the tube voltage and tube current of the tube ball. Firstly, for patients, the low line volume fluoroscopy mode further reduces the radiation dose by slightly increasing the tube voltage and tube current, reducing the radiation dose by more than 50% compared with the normal fluoroscopy mode (*p* < 0.001, 95% CI: 48.2–53.4%). A paired *t*-test was conducted to compare the radiation doses between the low-dose fluoroscopy mode and the normal fluoroscopy mode, confirming a statistically significant reduction in patient radiation dose (*t* = 12.45, *p* < 0.001). Increasing the tube current by 3–4 times the normal fluoroscopy radiation dose, see Table 1; secondly, for the operator, the low dose fluoroscopy mode can reduce the radiation dose of the operator by 25–34% (*p* < 0.01, 95% CI: 22.5–36.5%), as shown in Table 2 and Figure 3. An ANOVA test was performed to compare the operator radiation doses across different fluoroscopy modes, revealing significant differences (*F* = 18.76, *p* < 0.001).

**Table 1:** Variation of tube voltage, tube current, and patient radiation dose under different fluoroscopy modes.

**No**.	**Position**	**Indicator**	**Fluoroscopy 15 fps Low**	**Fluoroscopy 15 fps**	**Radiography 15 pps**
1	FPD: 10 cm, table height: 80 cm	Tube voltage (KV)	69	67	69
		Tube current (mA)	14.5	13	200
		Dose rate (mGy/min)	6.65	13.2	53.9
2	FPD: 10 cm, table height: 90 cm	Tube voltage (KV)	70	68	70
		Tube current (mA)	15.2	13.3	200
		Dose rate (mGy/min)	5.6	10.8	41
3	FPD: 10 cm, table height: 100 cm	Tube voltage (KV)	71	69	70
		Tube current (mA)	16.2	14.3	226
		Dose rate (mGy/min)	4.7	9.2	36.9
4	FPD: 20 cm, table height: 80 cm	Tube voltage (KV)	70	69	70
		Tube current (mA)	15.4	13.3	200
		Dose rate (mGy/min)	8.23	15.2	59
5	FPD: 20 cm, table height: 90 cm	Tube voltage (KV)	71	69	70
		Tube current (mA)	16.6	14.3	226
		Dose rate (mGy/min)	6.7	12.6	50
6	FPD: 20 cm, table height: 100 cm	Tube voltage (KV)	72	69	70
		Tube current (mA)	16.8	14.5	236
		Dose rate (mGy/min)	6.4	12	50
7	FPD: 30 cm, table height: 80 cm	Tube voltage (KV)	71	68	70
		Tube current (mA)	16.2	13.9	205
		Dose rate (mGy/min)	11.6	21.6	83.5
8	FPD: 0 cm, table height: 100 cm	Tube voltage (KV)	68	67	68
		Tube current (mA)	13.9	12.5	200
		Dose rate (mGy/min)	4.4	8.9	37
9	FPD: 0 cm, table height: 110 cm	Tube Voltage (KV)	70	67	69
		Tube current (mA)	15	13	200
		Dose rate (mGy/min)	3.94	7.4	30.4

The table provides a detailed comparison of technical parameters (tube voltage, tube current) and patient radiation dose across different fluoroscopy modes and bed heights.

**Table 2:** Variation of operator radiation dose under different fluoroscopy modes.

**Mode**	**Lens (mGy)**	**Thyroid (mGy)**	**Chest (mGy)**	**Gonads (mGy)**	**Lower Limbs (mGy)**
Fluoroscopy 15 fps	0.72 ± 0.31	1.13 ± 0.47	1.45 ± 0.62	4.07 ± 1.29	3.74 ± 1.05
Fluoroscopy 15 fps Low	0.47 ± 0.17	0.70 ± 0.27	0.89 ± 0.36	2.41 ± 0.72	2.33 ± 0.69

The table compares the operator radiation dose across different body parts (lens, thyroid, chest, gonads, and lower limbs) in normal and low-dose fluoroscopy modes.

### 3.2 Change of radiation dose under different bed plate height and different visual fields

To further explore the change of radiation dose between patient and operator after the change of bed plate and detector height, bed height, 80, 90, 100, and 110 cm, respectively and 10 cm (0 cm), 10, 20, and 30 cm, the radiation value of patient and operator at 90 and 10 cm were relatively small: direct ray 19.6 mGy/min (*p* < 0.05, 95% CI: 18.2–21.0 mGy/min), scattered ray 5.3 mSv/H (*p* < 0.05, 95% CI: 4.8–5.8 mSv/h), as shown in Tables 3, 4. Our results revealed significant differences in radiation doses across different bed heights and detector combinations (*p* < 0.001). An ANOVA test was conducted to compare the radiation doses across different bed heights and detector heights, showing significant differences (*F* = 24.89, *p* < 0.001).

**Table 3:** Changes in patient radiation measurement (mGy/min) at different bed heights and detector combinations.

**Bed height (cm)**	**0 cm**	**10 cm**	**20 cm**	**30 cm**
80 cm	-	24.2	29.3	43.0
90 cm	-	19.6	23.9	-
100 cm	14.7	17.1	12.0	-
110 cm	13.2	-	-	-

The table shows the patient radiation dose at various bed heights (80, 90, 100, and 110 cm) and detector heights (0, 10, 20, and 30 cm). The lowest radiation dose was observed at a bed height of 100 cm with a detector height of 20 cm.

**Table 4:** Changes in surgeon radiation measurement (mSv/h) at different bed heights and detector combinations.

**Bed height (cm)**	**0 cm**	**10 cm**	**20 cm**	**30 cm**
80 cm	-	4.8	4.3	4.3
90 cm	-	5.3	5.2	-
100 cm	6.0	5.5	5.8	-
110 cm	5.8	-	-	-

The table presents the operator radiation dose at various bed heights (80, 90, 100, and 110 cm) and detector heights (0, 10, 20, and 30 cm). The radiation dose to the surgeon was relatively low at bed heights of 80 cm to 90 cm.

### 3.3 Relationship between visual field size and radiation dose

**Figure 4:**
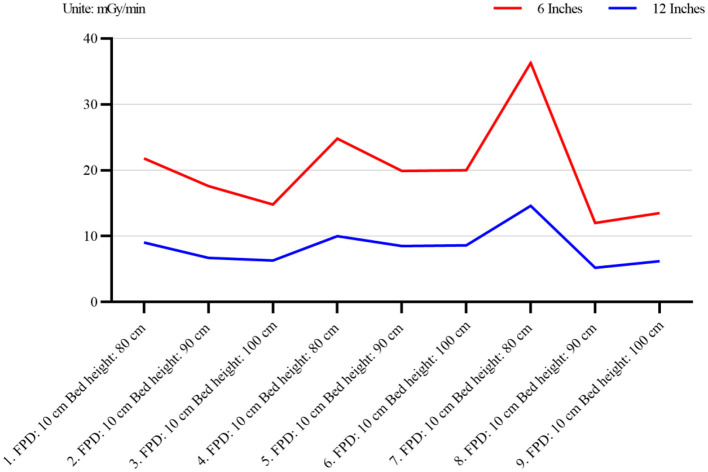
Radiation dose changes in patients at different visual field sizes (6 × 6 inches and 12 × 12 inches). The figure demonstrates the relationship between visual field size and patient radiation dose. As the visual field size increases, the patient's radiation dose also increases. The blue and white coordinates represent the radiation dose under the 6-inch and 12-inch fields, respectively.

In interventional surgery, different visual field sizes will be selected according to the needs of the operator. To clarify the radiation dose change when the size of the visual field changes, the radiation measurement changes of the patient and the operator were measured under the bed height and the detector fixation. When the radiation dose change curve of the patient becomes larger, the radiation dose also increases (*p* < 0.01, 95% CI: 12.5–18.7% increase per inch of visual field size), as shown in Figure 4. A linear regression analysis confirmed a significant positive correlation between visual field size and patient radiation dose (*R*^2^= 0.89, *p* < 0.001). A paired *t*-test was performed to compare the radiation doses between the 6 × 6 inches and 12 × 12 inches visual fields, revealing a significant increase in patient radiation dose (*t* = 8.34, *p* < 0.001).

### 3.4 Change of the operator's radiation dose after the application of protective facilities

**Figure 5:**
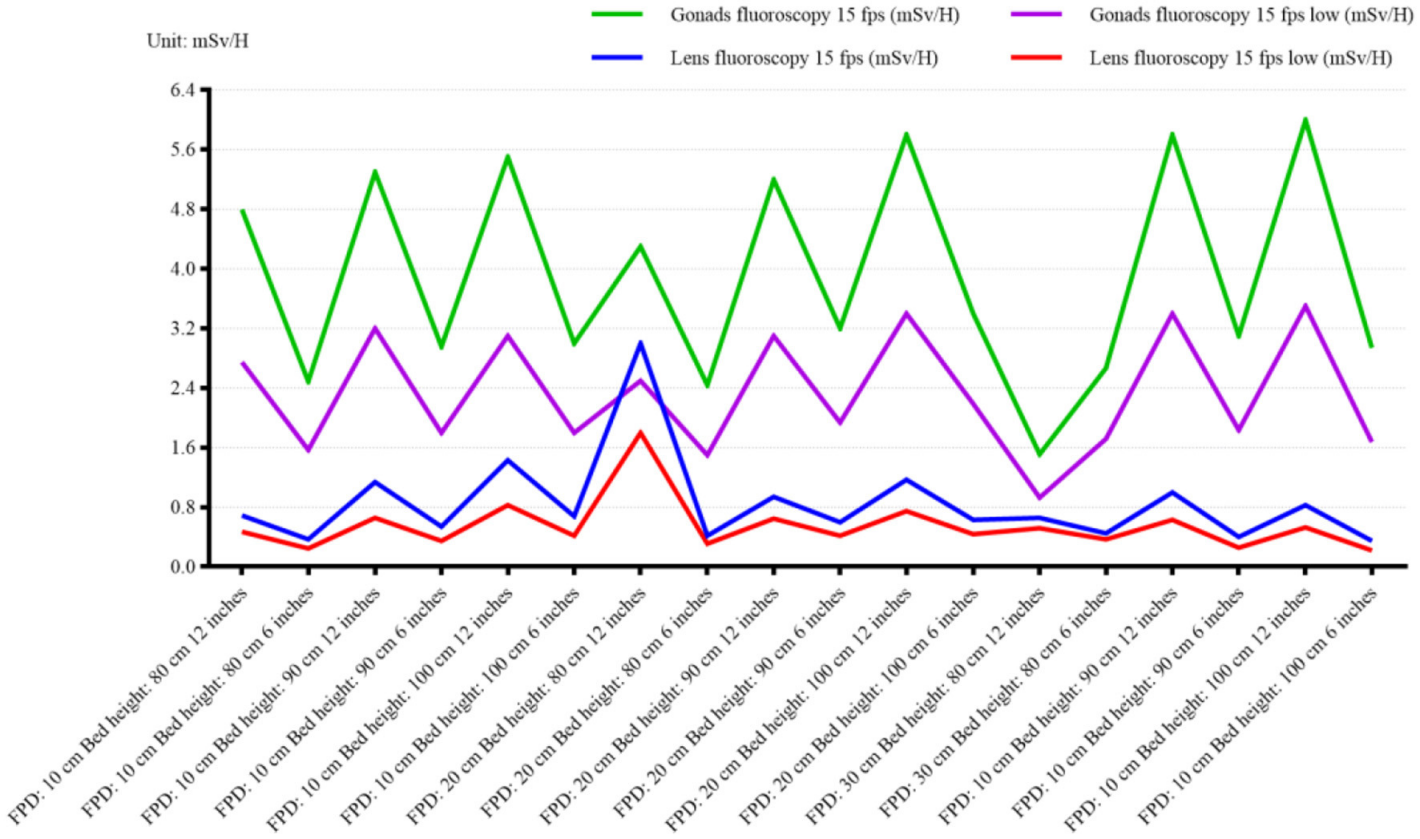
Surgical radiation dose changes under different visual field sizes, focusing on gonad and lens measurements in normal fluoroscopy mode (15 fps) and low-dose fluoroscopy mode (15 fps). The blue coordinates represent the radiation dose under the 6-inch field, and the white coordinates represent the radiation dose under the 12-inch field. The figure highlights the reduction in operator radiation dose with increasing visual field size, particularly for sensitive areas such as the gonads and lens.

**Figure 6:**
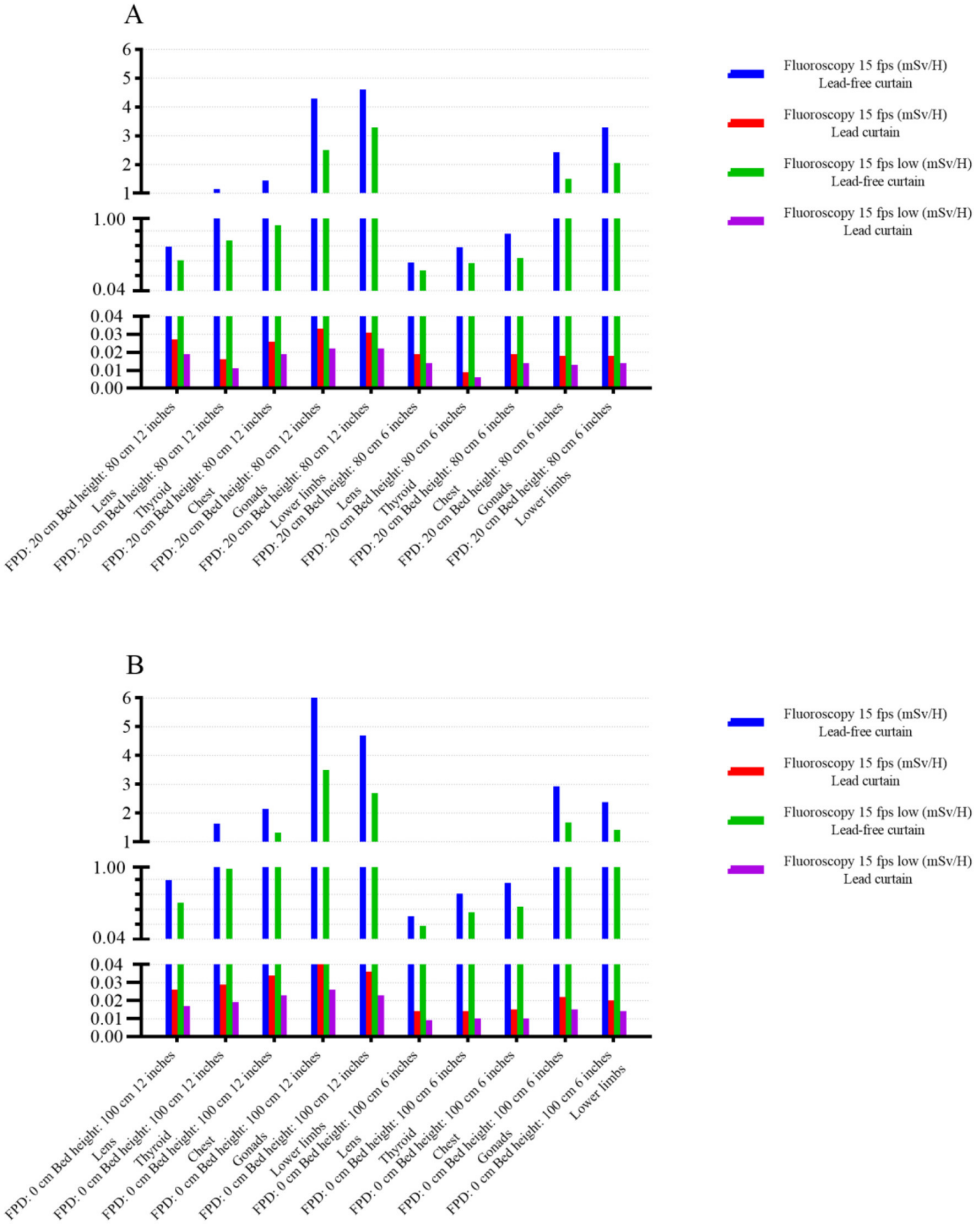
Comparison of operative radiation dose before and after the application of protective facilities (**A**: FPD 20 cm, bed height 80 cm; **B**: FPD 0 cm, bed height 100 cm). The figure shows the significant reduction in operator radiation dose after the application of protective devices such as lead curtains and shields. The blue and white coordinates represent the radiation dose under the 6-inch and 12-inch fields, respectively.

With the increase of interventional surgery, the radiation time of each operator is increasing. Currently, lead curtain and baffle shield are routinely applied in interventional surgery. We found that the application of protective measures could reduce the radiation dose by at least 10 times, as shown in Figures 5, 6. A paired *t*-test was conducted to compare the operator radiation doses before and after the application of protective facilities, showing a significant reduction (*t* = 15.67, *p* < 0.001).

## 4 Discussion

Our findings provide actionable insights for refining radiation safety protocols in interventional radiology. The demonstrated 50.8% reduction in patient radiation dose through low-dose fluoroscopy mode (Table 1) aligns with recent calls for dose optimization in guidelines such as the IAEA Safety Standards Series No. SSG-46. However, our study goes beyond previous research by systematically evaluating the combined effects of multiple DSA parameters, including bed plate height, detector height, and field of view size, on radiation dose.

Previous studies have primarily focused on individual parameters, such as low-dose fluoroscopy modes or the use of protective equipment, without considering the interplay between multiple variables. For patients, radiation is divided into two parts: fluoroscopy and contrast examination. Asmundo et al. (13) analyzed the radiation dose of patients receiving lower limb artery and aortic cavity intervention and found that the contrast time was much lower than the fluoroscopy time, but the radiation dose was much higher than the fluoroscopic radiation dose. And other studies have shown that the main radiation dose in interventional examination and treatment comes from intraoperative contrast examination (14). In this experiment, it was found that the radiation dose of the patient and the operator is multiplied, but it may also lead to the decline of the image quality. In addition, the low dose mode can reduce the radiation dose by changing the tube voltage and current, as shown in Table 1 and Figure 3. Therefore, low-dose fluoroscopy mode can be applied in low-precision fluoroscopy-guided interventional surgery to reduce patient and operator radiation, such as intestinal surgery at a part of DSA guidance and puncture and drainage surgery at non-important sites.

In addition to the radiation dose of the fluoroscopy mode, it is also related to the technical parameters in different modes, such as the change of the frame, the Angle of photography, the size of the photography range, the control of the distance between the detector to the tube, the change of the field of view and the (15–17) of the collimator range adjustment. In this experiment, we explored the change of radiation dose after the change of visual field size and found that the radiation increased while the radiation dose decreased in the patient after the visual field increased, as shown in Figures 4, 5. This is because the images are locally enlarged after photography when, the copper filtration parameters of the X-line ball tube will change accordingly. After the image is locally enlarged, Changing the parameter (18, 19) of copper filtration in the X-line ball tube, Copper filtration of 0.1 mm thickness is mostly used in the case of small-field fluoroscopy, A portion of the tissue is placed outside the detector, Will cause a portion of the X-ray following group to be photographed outside the detector, The tissue value of X-ray in the irradiation area will decrease (20, 21), To compensate for the absence of this fraction of the X-rays, DSA will decrease the thickness of copper filtration and increase the number of X-rays, And reduce the purity of the X-rays, Leading to an increase in patient radiation dose and a reduction in operator radiation dose (22). At the same time, in order to ensure the image quality, the automatic exposure system will improve the SNR ratio and X-ray quality (23) by adjusting the thickness of the copper plate filter. During interventional surgery, the bed plate height and detector height will be adjusted according to the patient and the intraoperative situation, and the radiation dose will change during the adjustment. The changes of radiation dose with different bed plate and detector height changes are shown in Tables 3, 4. After the increase of the bed plate, the radiation dose of patients decreases and the radiation dose of the operator increases, which may be due to the increased distance from the X-ray to the mold after the tube ball, which leads to the decrease of the X-ray quality, but also leads to the increase of scattering rays. During the operation, the appropriate bed plate and detector height can be selected to reduce the radiation dose of the patient and the doctor. For example, when the technician remotely controls the contrast, he can appropriately raise the bed plate to reduce the radiation dose of the patient, and reduce the bed plate height to facilitate the operation and reduce the radiation dose of the operator.

The quality of the X-ray beam is a crucial factor in determining both the dose delivered to the patient and the image quality obtained. One of the key parameters used to characterize beam quality is the half-value layer (HVL), which represents the thickness of a material that attenuates the X-ray beam to half of its original intensity. The HVL is directly related to the energy spectrum of the X-ray beam, with higher-energy beams typically having a greater HVL.

In our study, we have carefully measured the HVL for the X-ray beams used in our experiments. These measurements were conducted using standard techniques and equipment to ensure accuracy. The results indicate that the HVL values obtained were within the expected range for the specific X-ray tube and filtration used.

The importance of HVL in dose and image quality cannot be overstated. A higher HVL generally results in deeper penetration of the X-rays through the patient's tissue, which can lead to increased dose to the patient. However, this increased penetration can also result in better image contrast and visualization of deeper structures, provided that the imaging system is properly calibrated.

Therefore, when interpreting our findings, it is important to consider the HVL and energy spectrum of the X-ray beam used. Variations in these parameters can significantly affect the dose delivered and the image quality obtained, which in turn can impact the diagnostic accuracy and clinical outcome. By carefully measuring and controlling these factors, we can optimize the balance between dose and image quality, ensuring that patients receive the safest and most effective diagnostic imaging possible.

With the DSA exposure, the interventional professionals were illuminated with (24) by a large amount of scattered rays. According to the (25) of the national occupational health standard Requirements for medical X-ray Diagnosis (GBZ130-2013), “the dose rate level on the test plane of interventional occupational personnel in the fluoroscopic protection area in interventional surgery should not be 400 uSv/h.” In this experiment, the radiation dose of gonads and chest was higher than the national standard before the use of protective measures, but significantly less than the national standard after the application of protective facilities, as shown in Figure 6. At present, the protective equipment has also produced qualitative change with the progress of interventional technology and DSA machine. After the correct application of protective equipment in this experiment can reduce the radiation dose of the operator by more than 10 times. There have been many previous studies on the correct use method and necessity of protective equipment (25, 26). In addition, with the development of DSA machine technology, the application of more advanced vascular subtraction can further reduce the radiation dose. While a study demonstracted that a reduction the radiation dose of the hepatic artery chemoembolization by 52% (27) after the dose investigation of different interventional procedures in 16 hospitals in 13 countries.

However, there are some limitations to this study. First, the experiment was based on a specific digital subtraction angiography device (Shimadzu Trinias 12) and supporting dosimetry instruments (Raysafe X2 and AT1123), whose technical parameters (such as tube voltage, tube current regulation range) may be different from other brands or models. Therefore, the applicability of the proposed optimization scheme in different equipment needs to be further verified. Secondly, the bed plate height (80–110 cm), detector height (0–30 cm) and field of view size (6 × 6 inches and 12 × 12 inches) set in the experimental conditions are based on specific clinical scenarios, and the parameters may need to be adjusted in actual surgery due to patient size, anatomical location or surgeon habits, which may affect the universality of dose optimization. In addition, although the plexiglass model used in the study can simulate the direct radiation of patients, it cannot completely reproduce the influence of real patient tissue heterogeneity and dynamic physiological activities on radiation scattering. Finally, differences in the standardization of protective facilities (such as lead curtains and partitions) and operational processes in different medical institutions may interfere with the practical application of the research conclusions. Future studies need to verify the universality of the parameter optimization scheme under multi-center and multi-device conditions, and include real patient data to improve clinical guidance value. While this study provides valuable insights into the reduction of scattered radiation through optimized DSA parameters and protective measures, it has certain limitations. Specifically, we did not quantify the angular distribution and energy dependence of scattered radiation, which are critical for a more comprehensive analysis of radiation safety. Future studies should incorporate detailed measurements of scatter radiation at various angles and energy levels to better understand its behavior and further optimize radiation protection strategies.

While this study provides critical insights into optimizing DSA parameters for radiation safety, several avenues warrant further exploration (28, 29)]. First, multi-center trials are needed to validate the generalizability of our findings across diverse DSA systems (Siemens, Philips) and clinical workflows (30, 31). Second, incorporating real patient data—accounting for tissue heterogeneity, body mass index variations, and dynamic physiological movements (respiration)—would enhance the clinical relevance of dose optimization models. Third, the integration of artificial intelligence (AI) for real-time parameter adjustment, such as adaptive frame rate control or automated collimator positioning, could further minimize radiation exposure while maintaining procedural efficacy. Additionally, the development of lightweight, non-lead protective materials with improved ergonomics may address current barriers to consistent shielding compliance. Finally, longitudinal studies assessing the long-term health outcomes of optimized DSA protocols (reduced cancer incidence in operators) are essential to quantify the public health impact of these interventions.

## 5 Conclusion

Based on the above findings, the low dose pattern can significantly reduce radiation in patients and patients, In the image quality requirements of interventional surgery, low dose mode can be selected; Most of the time in fluoroscopic mode, At this time, both the patient and the operator received the X-ray radiation, The parameters of 90–100 cm from the ground and 10–20 cm from the patient can be selected for surgery to reduce radiation; For interventional surgery, The op cocoa leaves the operating room, Remote contrast was performed by a technician in the control room, To reduce the radiation dose received by the operator, (21), The height of the bed plate can be appropriately raised to increase the distance from the patient to the tube ball and then reduce the radiation dose. As a highly controllable adjustment parameter of the surgeon, the appropriate visual field size (22) should be selected according to the size of the subject and the area of interest. Finally, the correct use of protective devices can significantly reduce the radiation dose of the surgeon, and the doctors' protection awareness should be continuously strengthened and the protective measures are implemented. To enhance the comprehensiveness of radiation safety assessments, future research should focus on quantifying the angular distribution and energy dependence of scattered radiation. This will provide a more detailed understanding of scatter behavior and enable the development of more effective radiation protection protocols.

## Data Availability

The original contributions presented in the study are included in the article/supplementary material, further inquiries can be directed to the corresponding author.

## References

[1] Alexander HC Nguyen CH Chu MJJ Tarr GP Han CH Thomas RH. Transarterial radioembolization for hepatic metastases of pancreatic adenocarcinoma: a systematic review. J Vasc Interv Radiol. (2022) 33:1559–69.e2. 10.1016/j.jvir.2022.08.031 36084842

[2] Johnson DR Kyriou J Morton EJ Clifton A Fitzgerald M Macsweeney E. Radiation protection in interventional radiology. Clin Radiol. (2001) 56:99–106. 10.1053/crad.2000.0640 11222065

[3] Tsapaki V Ahmed NA AlSuwaidi JS Beganovic A Benider A BenOmrane L. Radiation exposure to patients during interventional procedures in 20 countries: initial IAEA project results. AJR Am J Roentgenol. (2009) 193:559–69. 10.2214/AJR.08.2115 19620457

[4] Ormiston JA Serruys PWS. Bioabsorbable coronary stents. Circ Cardiovasc Interv. (2009) 2:255–60. 10.1161/CIRCINTERVENTIONS.109.859173 20031723

[5] Kuon E Schmitt M Dahm JB. Significant reduction of radiation exposure to operator and staff during cardiac interventions by analysis of radiation leakage and improved lead shielding. Am J Cardiol. (2002) 89:44–9. 10.1016/s0002-9149(01)02161-0 11779521

[6] Huh HD Kim JH Kim SJ Yoo JM Seo SW. The change of lacrimal gland volume in Korean patients with thyroid-associated ophthalmopathy. Korean J Ophthalmol. (2016) 30:319–25. 10.3341/kjo.2016.30.5.319 27729751 PMC5057007

[7] Cho JH Kim JY Kang JE Park PE Kim JH Lim JA. A study to compare the radiation absorbed dose of the C-arm fluoroscopic modes. Korean J Pain. (2011) 24:199–204. 10.3344/kjp.2011.24.4.199 22220241 PMC3248583

[8] Lubis LE Bayuadi I Pawiro SA Ng KH Bosmans H Soejoko DS. Optimization of dose and image quality of paediatric cardiac catheterization procedure. Phys Med. (2015) 31:659–68. 10.1016/j.ejmp.2015.05.011 26050060

[9] Livingstone RS Varghese A. A simple quality control tool for assessing integrity of lead equivalent aprons. Indian J Radiol Imaging. (2018) 28:258–62. 10.4103/ijri.IJRI_374_17 30050253 PMC6038217

[10] Koirala N McLennan G. Mathematical models for blood flow quantification in dialysis access using angiography: a comparative study. Diagnostics. (2021) 11:1771. 10.3390/diagnostics11101771 34679469 PMC8534972

[11] Alizadeh LS Gyánó M Góg I Szigeti K Osváth S Kiss JP. Initial experience using digital variance angiography in context of prostatic artery embolization in comparison with digital subtraction angiography. Acad Radiol. (2023) 30:689–97. 10.1016/j.acra.2022.05.007 35688786

[12] Bosowska J Modlinska S Pekala T Szydło F Cebula M. Impact of monoplane to biplane angiography upgrade on diagnostic angiography procedures: a retrospective cross-sectional study. Phys Med. (2022) 98:40–4. 10.1016/j.ejmp.2022.04.011 35489130

[13] Asmundo L Rizzetto F Srinivas Rao S Sgrazzutti C Vicentin I Kambadakone A. Dual-energy CT applications on liver imaging: what radiologists and radiographers should know? A systematic review. Abdom Radiol. (2024) 49:3811–23. 10.1007/s00261-024-04380-y 38811447

[14] Smith-Bindman R Kang T Chu PW Wang Y Stewart C Das M. Large variation in radiation dose for routine abdomen CT: reasons for excess and easy tips for reduction. Eur Radiol. (2024) 34:2394–404. 10.1007/s00330-023-10076-6 37735276 PMC10957641

[15] Wijma IN Casal RF Cheng GZ Einsiedel PF Fantin A Hall DJ. Radiation principles, protection, and reporting for interventional pulmonology: a world association of bronchology and interventional pulmonology white paper. Respiration. (2024) 103:707–22. 10.1159/000540102 39033746 PMC11548093

[16] Huang W Lu J Chen KM Wu ZY Wang QB Liu JJ. Preliminary application of 3D-printed coplanar template for iodine-125 seed implantation therapy in patients with advanced pancreatic cancer. World J Gastroenterol. (2018) 24:5280–87. 10.3748/wjg.v24.i46.5280 30581276 PMC6295836

[17] Hoeng L Exeli AK Krombach GA Schwandner T Agolli L Habermehl D. Very pronounced bowel sparing during radiation therapy for anal carcinoma using a natural spacer (Myoma) - a case report. Radiat Oncol. (2024) 19:145. 10.1186/s13014-024-02530-6 39407281 PMC11479546

[18] Nocetti D Villalobos K Wunderle K. Physical image quality metrics for the characterization of X-ray systems used in fluoroscopy-guided pediatric cardiac interventional procedures: a systematic review. Children. (2023) 10:1784. 10.3390/children10111784 38002875 PMC10670102

[19] Slotman BJ Clark MA Özyar E Kim M Itami J Tallet A. Clinical adoption patterns of 0.35 Tesla MR-guided radiation therapy in Europe and Asia. Radiat Oncol. (2022) 17:146. 10.1186/s13014-022-02114-2 35996192 PMC9396857

[20] Nessipkhan A Matsuda N Takamura N Oriuchi N Ito H Awai K. The influence of revised ordinance on radiation protection management in Japanese hospitals: device deployment and involvement of radiology technologists. Jpn J Radiol. (2025) 43:117–28. 10.1007/s11604-024-01653-w 39340740

[21] Hong XL Loh YH Li DB Luan Y Zhang WB. Intracoronary administration of tenecteplase to prevent PCI-related myocardial infarction in patients with echo-attenuated coronary plaques: study protocol for a multicenter, prospective, randomized controlled trial. Trials. (2024) 25:794. 10.1186/s13063-024-08605-9 39587685 PMC11587588

[22] Wanke I Ewen K. Reduzierung der strahlenexposition für patienten und personal bei radiologischen interventionen am beispiel der coilembolisation. RoFo. (2006) 178:103–8. 10.1055/s-2005-858759 16392064

[23] Murthy V Maitre P Bakshi G Pal M Singh M Sharma R. Bladder adjuvant radiation therapy (BART): acute and late toxicity from a phase III multicenter randomized controlled trial. Int J Radiat Oncol Biol Phys. (2025) 121:728–36. 10.1016/j.ijrobp.2024.09.040 39353477

[24] GBD 2021 US Burden of Disease and Forecasting Collaborators. Burden of disease scenarios by state in the USA, 2022-50: a forecasting analysis for the Global Burden of Disease Study 2021. Lancet. (2024) 404:2341–70. 10.1016/S0140-6736(24)02246-3 39645377 PMC11715278

[25] Guo L Bai Z Zhao D Wang Y. Multi-wavelength luminescent sensor by lanthanide complex doped amino-clay for visual ofloxacin detection. Spectrochim Acta A Mol Biomol Spectrosc. (2025) 330:125602. 10.1016/j.saa.2024.125602 39756135

[26] König AM Etzel R Thomas RP Mahnken AH. Personal radiation protection and corresponding dosimetry in interventional radiology: an overview and future developments. RoFo. (2019) 191:512–21. 10.1055/a-0800-0113 30703826

[27] de Ceuninck M Dujardin K Vanhaverbeke M Muyldermans P de Walle SV Pauwelyn M. Impact of X-ray protective drapes on operator and patient radiation exposure during cardiac catheterization. Catheter Cardiovasc Interv. (2025) 105:475–82. 10.1002/ccd.31329 39660857

[28] Malode A Makwana B Patel V Khadke S Parikh A Bagga A. Cardiotoxicity and peri-operative considerations in immune checkpoint inhibitor and chimeric antigen receptor T-cell therapy: a narrative review. Anaesthesia. (2025) 80:25–37. 10.1111/anae.16493 39776062

[29] Boudissa M Khoury G Franke J Gänsslen A Tonetti J. Navigation and 3D-imaging in pelvic ring surgery: a systematic review of prospective comparative studies. Arch Orthop Trauma Surg. (2024) 144:4549–59. 10.1007/s00402-024-05468-2 39068618

[30] Hakimi M Reeg A Celi de la Torre JA Jung G Reyes Del Castillo T Roos J. Lucerne milestone approach for benchmarking and education: Towards ultra-low dose endovascular aortic repair. J Vasc Surg Cases Innov Tech. (2024) 11:101705. 10.1016/j.jvscit.2024.101705 39844861 PMC11750475

[31] Wang E Manning J Varlotta CG Woo D Ayres E Abotsi E. Radiation exposure in posterior lumbar fusion: a comparison of CT image-guided navigation, robotic assistance, and intraoperative fluoroscopy. Glob Spine J. (2021) 11:450–7. 10.1177/2192568220908242 32875878 PMC8119907

